# Automatic segmentation of inconstant fractured fragments for tibia/fibula from CT images using deep learning

**DOI:** 10.1038/s41598-023-47706-4

**Published:** 2023-11-22

**Authors:** Hyeonjoo Kim, Young Dae Jeon, Ki Bong Park, Hayeong Cha, Moo-Sub Kim, Juyeon You, Se-Won Lee, Seung-Han Shin, Yang-Guk Chung, Sung Bin Kang, Won Seuk Jang, Do-Kun Yoon

**Affiliations:** 1 Department of Medical Device Engineering and Management, College of Medicine, Yonsei University, Seoul, Republic of Korea; 2 Department of Orthopedic Surgery, University of Ulsan, College of Medicine, Ulsan University Hospital, Ulsan, Republic of Korea; 3 Industrial R&D Center, KAVILAB Co. Ltd., Seoul, Republic of Korea; 4 Department of Orthopedic Surgery, Yeouido St. Mary’s Hospital,, College of Medicine, The Catholic University of Korea, Seoul, Republic of Korea; 5 Department of Orthopedic Surgery, Seoul St. Mary’s Hospital, College of Medicine, The Catholic University of Korea, Seoul, Republic of Korea

**Keywords:** Musculoskeletal system, Biomedical engineering, Medical imaging, Therapeutics

## Abstract

Orthopaedic surgeons need to correctly identify bone fragments using 2D/3D CT images before trauma surgery. Advances in deep learning technology provide good insights into trauma surgery over manual diagnosis. This study demonstrates the application of the DeepLab v3+ -based deep learning model for the automatic segmentation of fragments of the fractured tibia and fibula from CT images and the results of the evaluation of the performance of the automatic segmentation. The deep learning model, which was trained using over 11 million images, showed good performance with a global accuracy of 98.92%, a weighted intersection over the union of 0.9841, and a mean boundary F1 score of 0.8921. Moreover, deep learning performed 5–8 times faster than the experts’ recognition performed manually, which is comparatively inefficient, with almost the same significance. This study will play an important role in preoperative surgical planning for trauma surgery with convenience and speed.

## Introduction

Semantic segmentation is a core technology used to solve the challenges in the field of computer vision. Semantic segmentation can generate segmented images based on a pixel-based classification^1–3^. Classes (index, location, and area) are defined as units of pixels in the digital image. The development of deep convolutional neural networks (CNNs) has led to a high level of segmentation that can classify different instances in the same class^4–10^. Consequently, semantic segmentation shows powerful performance in applications such as automatic driving and the medical field for specific purposes^11^. Several studies have been conducted to improve the semantic segmentation performance^12–16^. The current popular algorithms for semantic segmentation include FCN, SegNet, PSPNet, DeepLab, and UNet^17^. In the DeepLab framework (DeepLab v1), atrous convolution was used in combination with CNN for semantic segmentation. To optimize performance, DeepLab v2 added a new model, atrous spatial pyramid pooling (ASPP), which utilized atrous convolution to get multi-scale information and reduced computation instead of fully connection layer. And DeepLab v3 improved the ASPP model with one 1 × 1 convolution and three 3 × 3 convolution^17^. This framework is a generic framework which can be applied to any network, such as VGG and ResNet. For DeepLab v3, a simple and efficient decoder model was designed to improve segmentation results. The FCN has the advantage becoming full convolutional layers without connected layers; however, it shows the low accuracy of the feature map with heavy GPU computation^17^. The SegNet which is the first symmetric network has a weak point as slow speed^17^. Although the UNet is suitable for object detection in small number of medical images, it is difficult to get the uniform standard of sub-sampling and up-sampling^17^. The PSPNet that uses a pyramid pooling module to identify prior information is good for identifying complex scenes, however, it has a limitation to the application of a specific model to the backbone^17^. Although the series of algorithms in DeepLab has only a weak point in the requirement of high GPU computation, Ruixin et al. mentioned that the series of algorithms in DeepLab provide a great choice for accurate delineation of specific margins in the medical image^17^. For these reasons, we considered the proper model as the latest version in the DeepLab series to perform segmentation on irregular and complex medical images. Recently, DeepLab v3 + , introduced by Google in 2018, has shown high performance in semantic segmentation^18^. The DeepLab v3+ is a model including Atrous Separable Convolution which is a combination of Depthwise Separable Convolution and Atrous Convolution^19–21^. An advantage of depth-separable convolution is that an outcome similar to that of the conventional convolution method can be obtained with dramatically decreased computational complexity. DeepLab v3+ uses an encoder-decoder structure and a backbone as a residual neural network (ResNet) model, which was first developed by Microsoft^18–22^. The signature specification of the ResNet is a skip connection. The skip connection (shortcut connection) in the ResNet model compensates for the vanishing gradient problem^23,24^. DeepLab v3+ is one of the strongest models for solving the segmentation challenge and has been developed to perform semantic segmentation for complex images^23^. Since the specification of semantic segmentation is based on the image and can provide precise information for a specific area on that image, the application of semantic segmentation in the medical field is an advantage^25–36^. Many clinical fields require accurate segmented images from digital imaging and communications in medicine (DICOM) for diagnostics, planning, and simulation^29–36^. For example, a tumor region that is difficult to detect can be segmented from an image^17,37–39^. A 3D vessel model can be reconstructed using a 2D segmented image to establish plans for approach and stent insertion^36^. Segmented bone areas from computed tomography (CT) images can be used to fabricate patient-specific instruments or simulate surgical processes^40^. However, segmented images are typically acquired manually or interactively using a dedicated tool^40–42^. Unfortunately, medical centers still perform image segmentation using these methods when required. In this case, successful image segmentation is time-consuming and requires trained practitioners^40–42^. Therefore, the effectiveness and usefulness of semantic segmentation are remarkable^25^.

Orthopedic trauma includes several fractures with various patterns and conditions. In particular, a highly complex comminuted fracture makes it difficult for surgeons to perform reduction and the operation is time-consuming^41–46^. Therefore, surgeons typically want to correctly identify bone fragments before surgery using 2D/3D CT images^41,43^. However, because it is difficult for surgeons are mostly hard to identify all fragments of the bone by comminuted fractures using only CT images and one color, they find unidentified fragments during the operation with an open approach^41^. Naturally, this type of fragment can be a critical factor in extending surgical time. The semantic segmentation of bone fragments can provide intuitive segmented results for each fragment and insight into a preoperative surgical plan for comminuted fractures^33–35^. In this study, although we developed three candidate deep learning models based on ResNet using the DeepLab v3+ model as an encoder-decoder, the best deep learning model was applied to perform automatic segmentation of the fracture fragment from the CT image. In particular, the network model was designed to exhibit high efficiency at high speeds using a small amount of data. We used data from only 105 patients (11,891,000 image sets with data augmentation) who underwent trauma surgery for the tibia and fibula as training data and 50 CT image series were used to test the model. This study aimed to apply the best deep learning model to the automatic segmentation of fragments of the fractured tibia and fibula from CT images and to evaluate its performance with respect to image analysis and clinical support.

## Results

### Results of the deep learning model training and analysis of data for training

**Figure 1:**
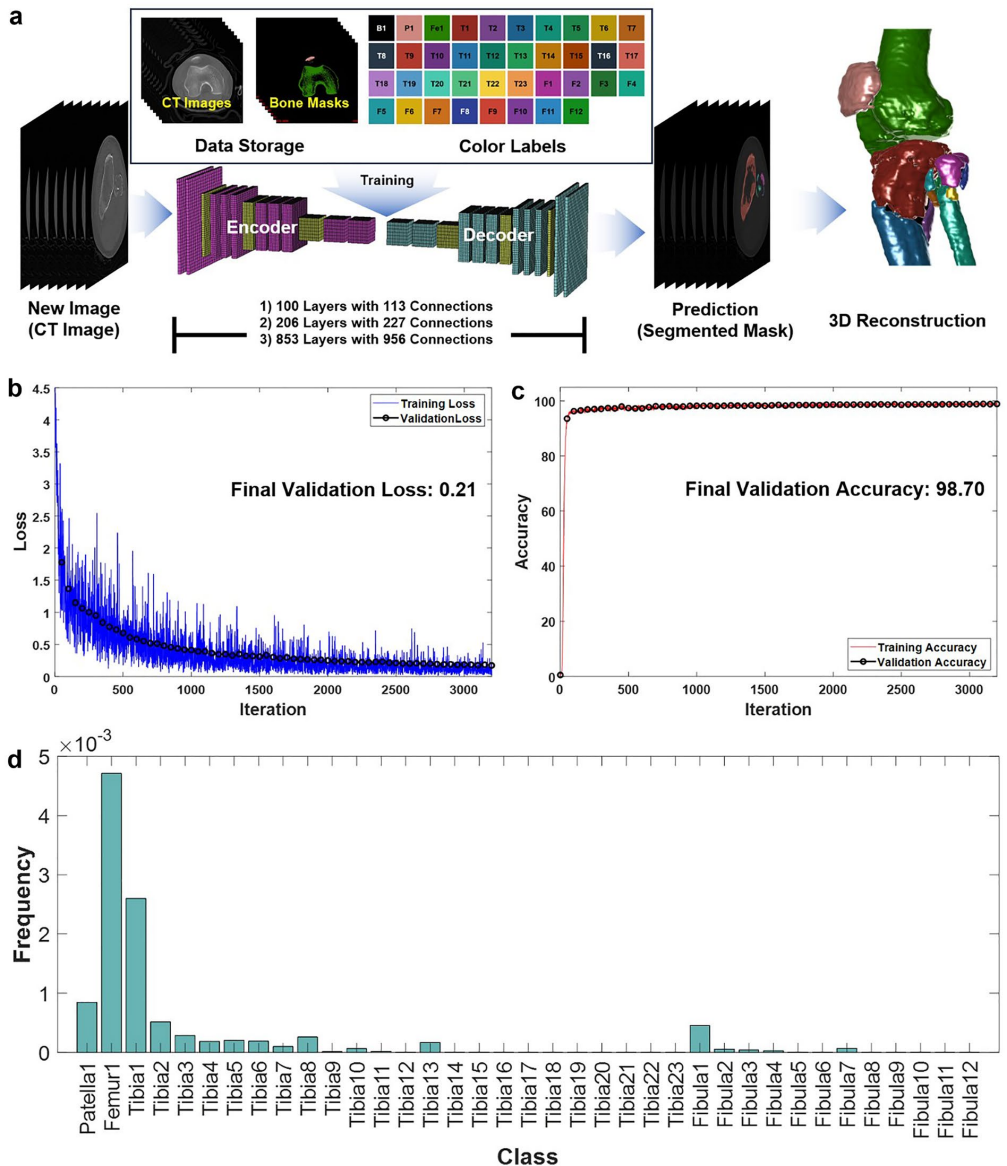
Overview of automatic segmentation of the fracture fragments from the CT images using deep learning and the best performance of the training results. (**a**) The data storage and the appointed color label and the brief specifications of the designed deep learning models. (**b**) The best performance of final validation loss by the training (from the second model: 206 layers with 227 connections) and, (**c**) The best output of final validation accuracy by the training (from the second model: 206 layers with 227 connections). (**d**) The frequency level for the counted pixel according to class.

Figure 1 shows the overall method for performing automatic segmentation using the deep learning model and the training results. As shown in Fig. 1a, the segmented image (bone mask image) data were prepared by the manual segmentation of 11,891,000 images with the appointed colors to train all three deep learning models. To apply the segmentation for fracture fragments in the tibia and fibula cases, 23 colors for the tibia and 12 colors for the fibula were labeled to the segmented data. The number of colors and orders were continuously added according to the appearance of more fragments during data preparation, and the final number and order were determined after the manual segmentation of all data. The dataset comprised both CT and bone mask images, which were arranged for data storage according to each series. Three candidate deep-learning models were used to perform the automatic segmentation of fracture fragments from the CT images. The models had 100, 206, and 853 layers with 113, 227, and 956 connections, respectively. All models were repeatedly trained to determine the best performance by optimizing the hyperparameters. The best validation loss and accuracy were found to be 0.21, and 98.70%, respectively, from the second model (206 layers with 227 connections; Fig. 1b and c). For the analysis of the training data, the number of pixels according to class was counted, and the frequency level (the counted pixels for each class/the counted pixels for all classes) for each class is shown in Fig. 1d. The frequency of the background (black) was removed from Fig. 1d because of the overwhelming difference between the frequencies of the background and the others.

### Performance evaluation for segmentation using deep learning

**Figure 2:**
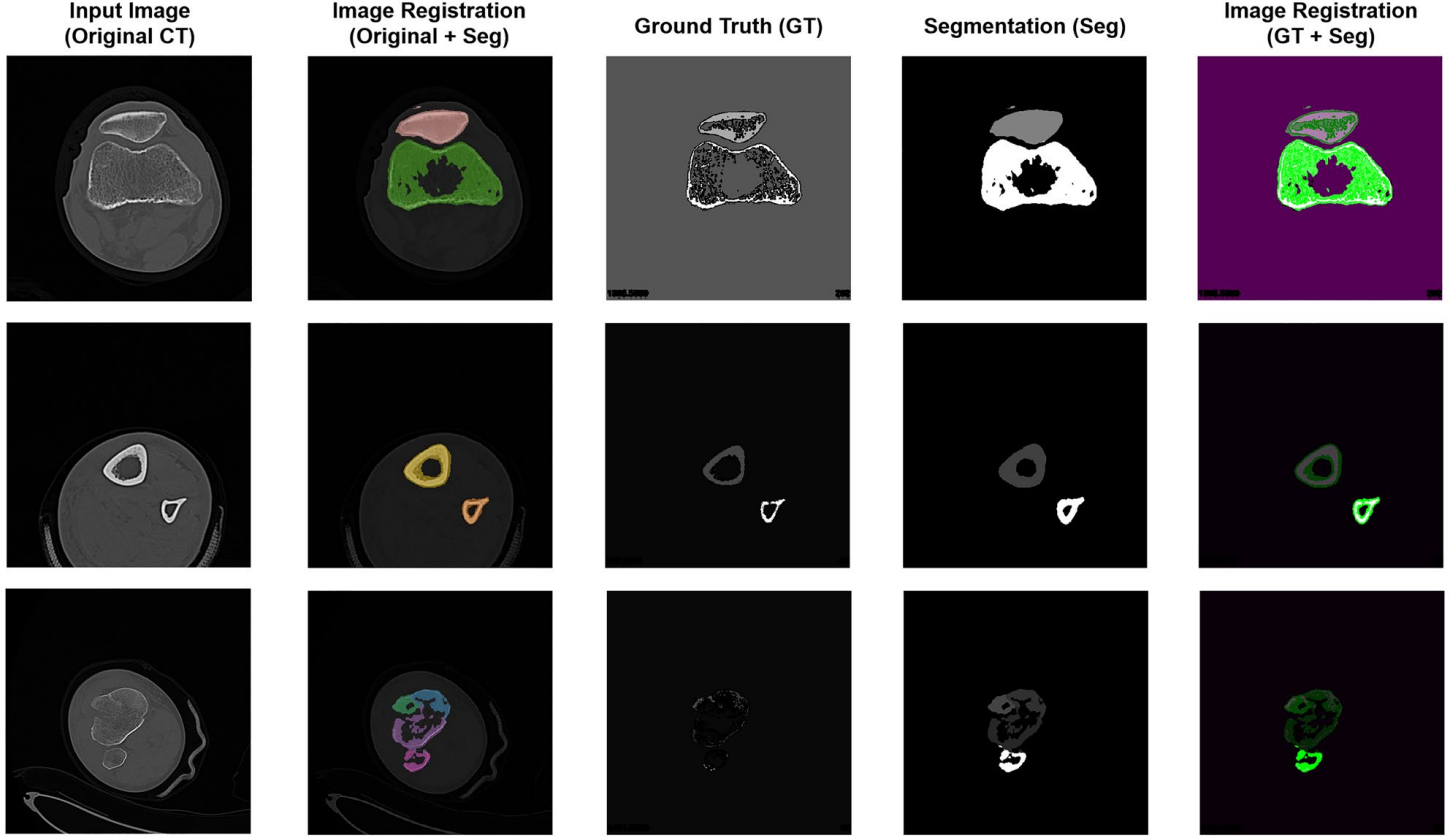
The partial results to describe the calculation process of the evaluation indicator for the deep learning model. The first column shows the original CT images as the input of deep learning. The registered images between the original CT images and the segmented mask by deep learning are shown in the second column. The third column and fourth column show the ground-truth images which are the bone masks by the manual segmentation and the segmented masks by the deep learning, respectively. And the registered images between the ground truth and the segmented mask are demonstrated in the last column.

To evaluate the actual performance of the segmentation, we investigated several indicators that can demonstrate the performance of deep learning and determined the best model for the automatic segmentation of fracture fragments from CT images. All indicators for the evaluation of the deep learning models were acquired by comparing the ground truth and segmented mask. The ground truth is a bone mask (Fig. 1a), which is an image manually masked by a human without the application of deep learning. The segmented image is automatically acquired by the deep learning model. By registering the two images, all indicators for the evaluation of the deep learning model were calculated. Figure 2 shows the partial results of the calculation process used to evaluate the performance of deep learning. The original CT images as the input of deep learning were presented in the first column. The second column shows the registered images between the original CT images and the segmented mask obtained using deep learning. The third and fourth columns show the ground truth and segmented masks obtained using deep learning, respectively. The last column shows the registered images between the ground truth and segmented masks obtained using deep learning. In the images in the last column, the white regions indicate well-matched regions between the two images. In contrast, the green regions show the regions predicted differently from the ground truth.

Through the process in Fig. 2, the summary results of the evaluation of all the deep learning models are presented in Table 1. The major indicators in Table 1 are the accuracy of the classified pixels, the intersection over union (IoU), and the boundary F1 (BF) score. Accuracy includes the ‘Global Accuracy’ and ‘Mean Accuracy’. The IoU has two indicators: the Mean IoU’ and ‘Weighted IoU. As shown in Table 2, Model 2 showed the best output from all indicators among the three models. And the three representative evaluation indicators (‘Accuracy, ‘IoU’ and ‘Mean BF Score’) according to the class for Model 2 are listed in Table 2. The accuracy in Table 2 is the ratio of correctly classified pixels in each class to the total number of pixels belonging to that class, according to the ground truth. The ‘IoU’ in Table 2 means the ratio of correctly classified pixels to the total number of pixels that are assigned that class by the ground truth and the model. Lastly, the ‘Mean BF Score’ in Table 2 is the BF score for each class, averaged over all images.

**Table 1:** Summary results for evaluating the performance of the three deep learning models.

	Global accuracy	Mean accuracy	Mean IoU	Weighted IoU	Mean BF score
Model 1	0.9783	0.7384	0.1572	0.9758	0.8210
Model 2	0.9892	0.8344	0.3049	0.9841	0.8921
Model 3	0.9868	0.7917	0.2584	0.9815	0.8654

*Global Accuracy* Ratio of correctly classified pixels to total pixels, regardless of class; *Mean Accuracy* Ratio of correctly classified pixels in each class to total pixels, averaged over all classes; *Mean IoU* Average intersection over union (IoU) of all classes; *Weighted IoU* Average IoU of all classes, weighted by the number of pixels in the class; *Mean BF Score* Average contour matching score for image segmentation.

**Table 2:** Results for performance of segmentation by the Model 2.

Class	Accuracy	IoU	Mean BF score	Class	Accuracy	IoU	Mean BF score
Background	0.9895	0.9894	0.9385	Tibia 17	0.2914	0.0178	0.2874
Patella	0.9967	0.5729	0.8800	Tibia 18	1.0000	0.0106	0.5515
Femur	0.9913	0.5520	0.9161	Tibia 19	0.9637	0.1981	0.8118
Tibia 1	0.9562	0.5227	0.8392	Tibia 20	1.0000	0.3301	0.7234
Tibia 2	0.8250	0.2944	0.6561	Tibia 21	1.0000	0.1988	0.7932
Tibia 3	0.8911	0.3173	0.6721	Tibia 22	0.8716	0.1644	0.6987
Tibia 4	0.9418	0.4024	0.6914	Tibia 23	0.5000	0.5000	1.0000
Tibia 5	0.9280	0.3190	0.7319	Fibula 1	0.9833	0.3604	0.8952
Tibia 6	0.9713	0.4850	0.7967	Fibula 2	0.9423	0.3611	0.8531
Tibia 7	0.9261	0.4166	0.7294	Fibula 3	0.9285	0.2044	0.7706
Tibia 8	0.8873	0.5333	0.8015	Fibula 4	0.9480	0.2804	0.7910
Tibia 9	0.9184	0.1800	0.6484	Fibula 5	0.9251	0.1654	0.8092
Tibia 10	0.9255	0.3874	0.797	Fibula 6	0.7196	0.2190	0.6195
Tibia 11	0.8553	0.4500	0.8005	Fibula 7	0.9786	0.4131	0.9061
Tibia 12	0.9163	0.2118	0.6368	Fibula 8	0.5910	0.1708	0.5914
Tibia 13	0.9703	0.5665	0.8187	Fibula 9	0.2000	0.0123	0.5411
Tibia 14	0.5279	0.1773	0.6072	Fibula 10	0.9873	0.1302	0.8452
Tibia 15	0.8551	0.1435	0.5201	Fibula 11	0.5510	0.1374	0.8447
Tibia 16	0.9116	0.1710	0.6185	Fibula 11	0.1429	0.0173	0.1522

*Accuracy* Ratio of correctly classified pixels in each class to the total number of pixels belonging to that class according to the ground truth. *IoU* Ratio of correctly classified pixels to the total number of pixels that are assigned that class by the ground truth and the model. *Mean BF Score* Boundary F1 score for each class, averaged over all images.

### Segmentation performance-based 3D reconstruction using CT series

The ultimate purpose of automatic segmentation of the fracture fragment is to visualize the 3D bone image, including the classified fracture fragments, using intuitive identifiers such as several colors. As the deep learning model can predict the bone mask for each CT image slice-by-slice, segmented masks can be generated for all CT images in the series.

**Figure 3:**
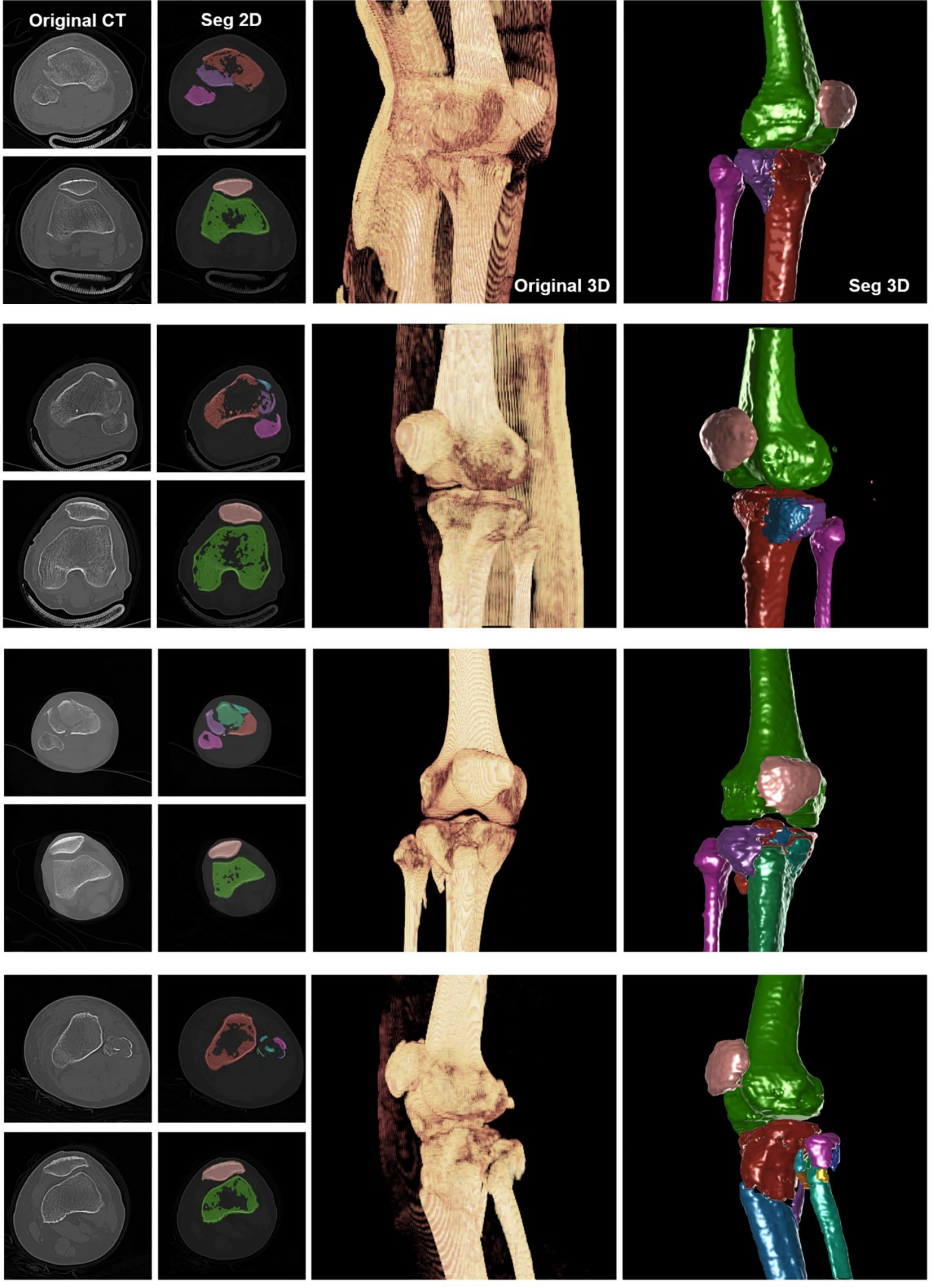
Representative 3D reconstructed images by automatic segmentation via deep learning. The first column includes the part of original computed tomography (CT) images in the CT series. The 2D registered images between the original CT images and the segmented mask by deep learning are demonstrated in the second column. The third column shows the 3D reconstructed bone image using the original CT images with the minimum threshold set for the Hounsfield Unit (HU) of the CT images. The last column shows the 3D reconstructed images using the segmented masks by deep learning. The colors according to the fragments are matched with the colors on the figure in the second column.

**Figure 4:**
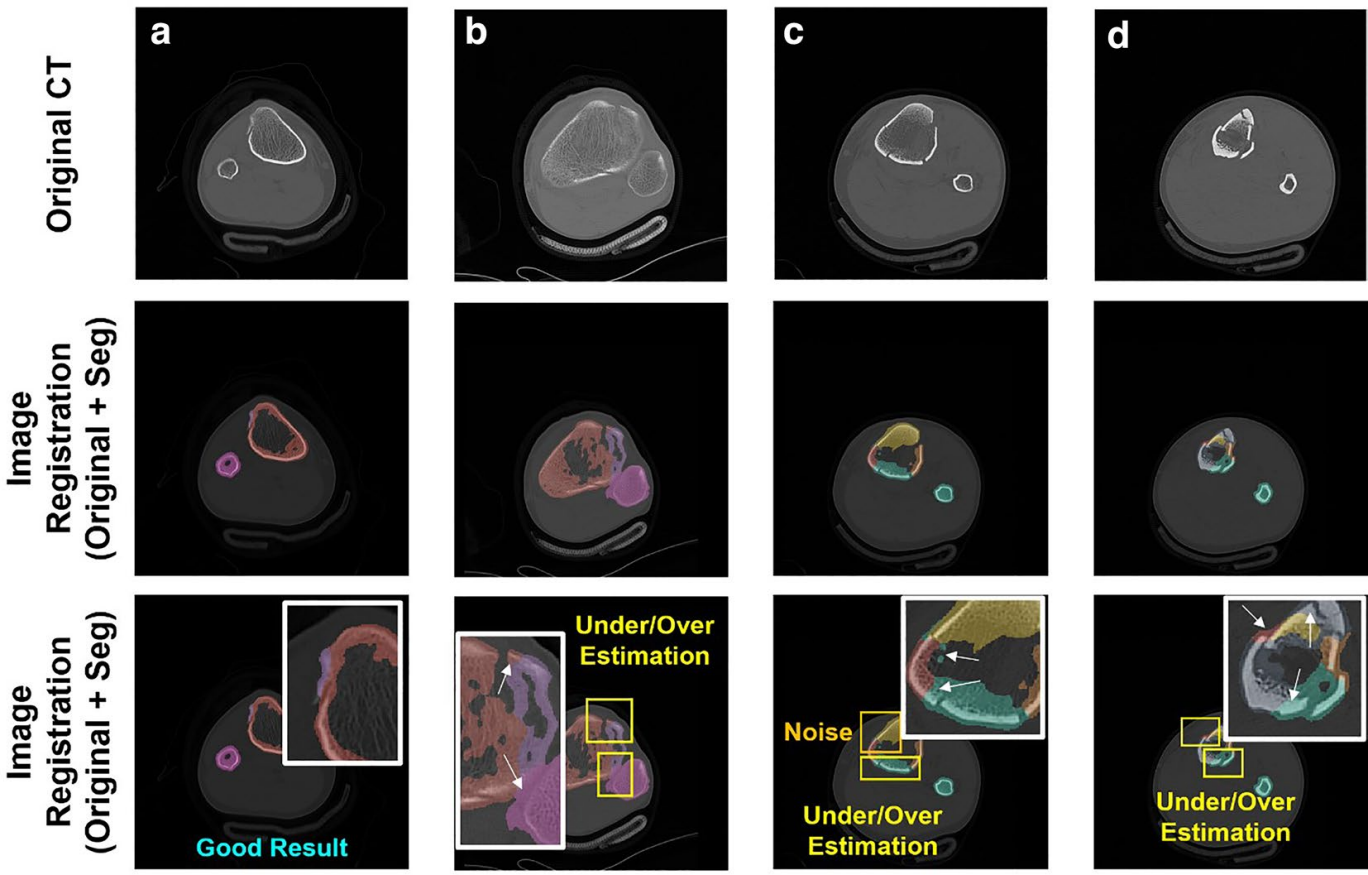
Representative low-quality results for the segmentation by deep learning due to under/over estimation (yellow boxes) and noise generation (orange box), detail points and the expanded view by the white arrows in the white boxes. (**a**) The good results for segmentation by deep learning. (**b**) Under estimation: violet mask, overestimation: red and pink masks. (**c**) Under estimation: red mask, overestimation: green mask, noise: green mask. (**d**) Under estimation: yellow and dark blue masks, overestimation: green and dark blue masks.

Figure 3 shows the representative segmentation performance for 3D reconstruction using Model 2. Of the 50 test cases, which were patient CT series including trauma fractures of the tibia or fibula, the case with the most fragments showed 10 fragments, except for the patella and femur. The cases with the least number of fragments showed only two fragments on the tibia or fibula. The results of the 3D reconstruction for the segmented fractured fragments are displayed from top to bottom in the order of fragments 2, 3, 7, and 10 in Fig. 3. The first and second columns show the original CT images and registered images between the original CT image and segmented masks, respectively. In the registered images, the slices involving the regions of the patella and femur, as well as the representative slice, which can show signature results for the segmentation of several fragments, were demonstrated. When orthopaedic surgeons normally examine CT images from a picture archiving and communication system (PACS), they find and confirm the original CT images and 3D reconstructed images like the figures in the third column of Fig. 3. A general 3D reconstructed bone image can be acquired using the minimum threshold set for Hounsfield Unit (HU) filtering for a region of interest (ROI) on the CT images. Although the 3D reconstruction view in PACS can provide the whole structure of the ROI as a 3D object, the fractured fragments are still difficult to correctly identify owing to several factors, such as the unclear boundary, monotone color, threshold abnormality caused by the low image quality, artifacts, and external devices. These types of problems are also observed from the figures in the third column of Fig. 3. However, the 3D reconstructed images using the segmented masks in the last column of Fig. 3 show a clearer boundary and definite shape for each fragment with a different color than the generally reconstructed 3D image in the third column. However, improvements were observed for several slices. The deep learning results in this study showed under-or overestimation of segmentation from some slices as one of the limitations. As shown in Fig. 4, parts of the regions for the color mask were assigned as incorrect regions. The first and second rows show the original CT images and the registered image between the original CT images and the segmented mask, respectively. The third row includes the diagnosis of the issues using several colored boxes. In normal cases, the color masks should be divided according to the discrete bone shape as a boundary between the fragments, as shown in Fig. 4a. When one color mask is overestimated, the other color masks are easily underestimated because each pixel should be filled with a dedicated mask. Figure 4b–d shows representative results for low-quality results by under/overestimation of deep learning for segmentation. The yellow boxes show regions that included an example of underestimation/overestimation. The white arrows in the white boxes indicate detailed points. Another limitation of deep learning results in this study is noise generation by the masks, as shown in the orange box in Fig. 4c. Although the noise caused by the mask is not a major issue in deep learning segmentation, it can decrease the quality of the final results. This issue is also observed in the second row of Fig. 2. Small red volumes were detected on the outer side of the main bone image; however, these volumes were not related to the fractured fragments.

### Clinical support ability

**Figure 5:**
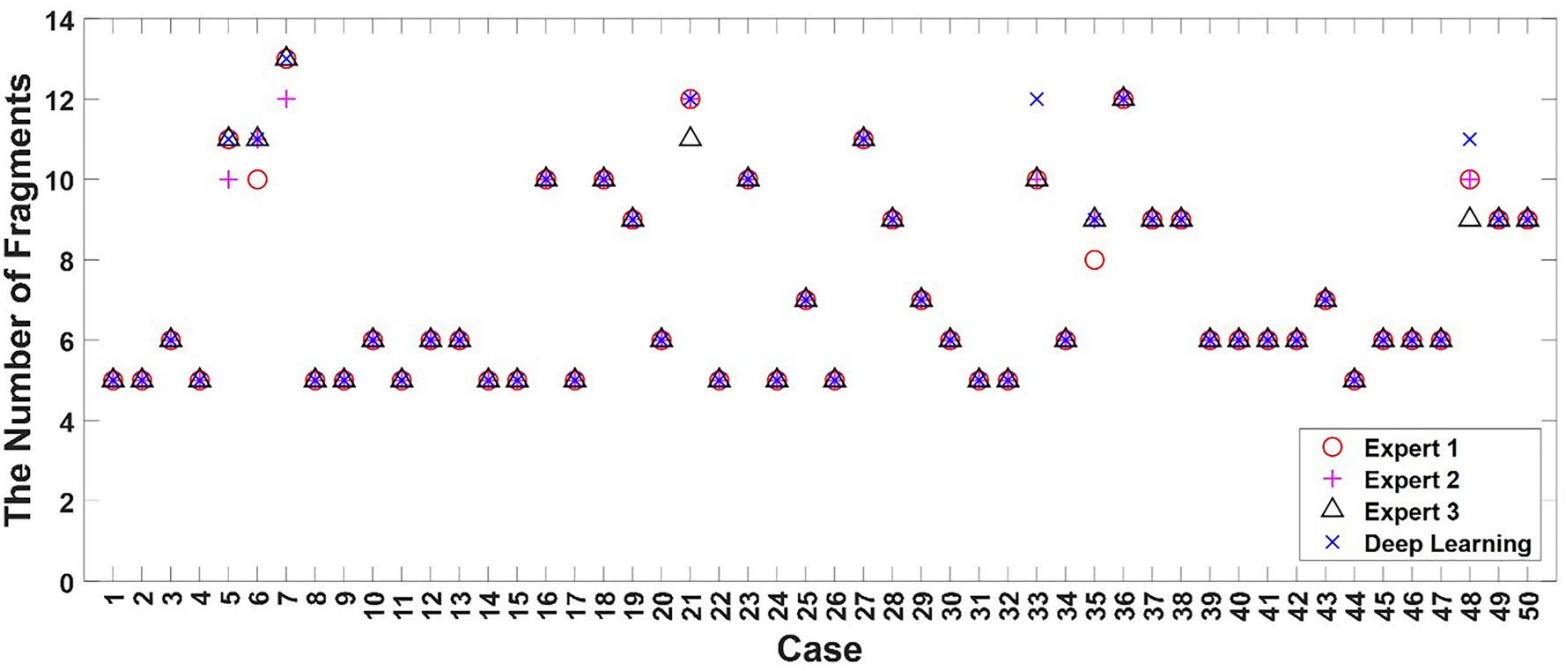
The counted number of fractured fragments by the three experts and deep learning for test 50 CT series (cases). The three experts identified the shape of the fragments and counted the number of the fragments using only 2D/3D CT images (red circle: Expert 1, pink cross: Expert 2, black triangle: Expert 3). The blue X marker shows the counted number of fractured fragments by the automatic segmentation using deep learning.

The reliability and fast acquisition of results are essential to obtain the actual assistance of deep learning for identifying fractured fragments before surgery. First, the purpose of automatic segmentation of the fractured fragment from the CT images was to correctly identify the status of the fragments, such as the number of fragments, shape of fragments, and boundaries between fragments. The accuracy of the shape of the fragments by deep-learning-based automatic segmentation is reported in Table 2. We also prepared 50 test cases to confirm the clinical support ability of deep-learning-based automatic segmentation instead of observing 2D/3D CT images for trauma surgery. Three experts, including data engineers who prepared the data set for this study and orthopaedic surgeons who provided the data set, manually recognized the fragments using only 2D/3D CT images and counted the number of fragments based on their approximate shapes. Figure 5 shows the status of the number of fragments counted by expert 1, 2, 3, and the deep learning model. The red circles, pink crosses, and black triangles represent the number of fragments counted from the 2D/3D CT images by experts 1, 2, and 3. Finally, the blue X marker indicates the number of fragments counted via automatic segmentation using deep learning.

We also checked the paired t-test to verify the significance of the results obtained by the experts and the results of deep learning (Table 3). The null hypothesis was that there would be no differences in the results between the experts and deep learning. There were no statistically significant differences between the human and deep-learning results (h = 0).

**Table 3:** Verification of significance of the results at Fig. 5 between by the experts and deep learning.

	Expert 1—deep learning	Expert 2—deep learning	Expert 3—deep learning
h	0	0	0
*p*-value	0.058	0.058	0.0959

Where h is test decision. If h-value is zero, two groups did not have any differences statistically.

The time required to identify the fractured fragments is another important factor affecting the clinical support ability of orthopaedic surgeons. When statistical significance showed no difference in the results between experts and deep learning automatic segmentation, deep learning was superior in terms of time cost. Since the manual segmentation per project takes more than 2 h to complete, the time to complete manual segmentation is actually not a comparison target with automatic segmentation by deep learning. Hence, we measured the time required by the expert to identify all fragments from the 2D/3D CT images (Fig. 5). This time was compared with the time required for automatic segmentation using deep learning. Naturally, deep learning provides a full 3D reconstructed image with all objects for the segmented fragments. However, experts recognized only the number of fractured fragments using 2D and 3D CT images. The deep learning finished segmenting all the fragments using 3D reconstruction within an average of 14 s (13.56 (standard deviation (std): 0.87) sec). However, the experts took a longer time to identify all fragments (Expert 1: 73.67 (std: 26.22) sec, Expert 2: 117.30 (std: 19.45) sec, Expert 3: 89.44 (std; 15.81) sec). In the simple case, the expert identified all fragments faster than deep learning in some cases. However, the experts took much more time to identify the fragments in almost all cases. Deep learning performs automatic segmentation without time fluctuations regardless of the complexity of the fracture pattern.

## Discussion

The key aspect of this study is how quickly and accurately deep learning provides intuitive 3D segmented images, as shown in the last column of Fig. 3. Normally, most surgeons identify fractured fragments using only 2D/3D CT images during diagnosis or preoperative surgical planning^40–46^. Moreover, although there are several dedicated software and 3D modeling tools, few orthopaedic surgeons perform manual segmentation with a long working time (approximately 2–3 h) before trauma surgery, except for research purposes or the fabrication of patient-specific devices^40–43^. The correct identification of the shape of the fractured fragments, their numbers, and clear boundaries between fragments before surgery provides strong insight into the reduction plan and the strategy for the use of the implant. In addition, this insight provides an opportunity to reduce the operation time, pain level, and bleeding volume^44,45^. Although advanced studies and performance improvements for deep learning, even additional data collection, are essential, deep learning in this study provided stable results very quickly (within an average of 14 s) when the input case involved a fracture within 12 fragments.

In this study, we used the training data, which focused on the fracture cases of the tibia and fibula. When we secured the additional CT image, including other fractured regions such as the patella, femur, and even pelvis, we could train the deep learning model as a transfer learning method by adding the class and its own color label. The reason for selecting trauma cases of the tibia and fibula as the first training case is that the tibia and fibula can create the most complex pattern for the fracture^41,42^. These are long bones, including the articular surface, and a sufficiently large volume that can generate many fragments.

In this study, the indicators for the performance of the deep learning model were reported as accuracy (global accuracy, mean accuracy), IoU (mean IoU, weighted IoU), and Mean BF score. Although accuracy is a general verification indicator of deep learning, the individual accuracy for each class cannot be distinguished from the global or mean accuracy since the counted number of pixels for the background is overwhelmingly large^18–25^. Instead, as shown in Table 2, deep learning provided many correct estimations (true positive + true negative) according to each class. Although IoUs have been reported to have relatively low values, this tendency is inevitable. As shown in Fig. 2, the ground truth shows a sparse mask in the spongy bone region. However, the segmented mask was dense with a thick ring in the spongy bone region. The external boundary of the mask followed the external shape of the original bone relatively well.

In the data preparation process, the ground truth image was drawn using a threshold set depending on the HU value of the CT image^29–35^. In this case, when the pixel has an HU value below the set threshold, the pixel of the ground truth does not cover the mask, despite the pixel in the region of the spongy bone. Although the segmented mask correctly covered the region of the spongy bone, the IoUs reported low values due to the sparse mask in the spongy bone region in the ground truth. Hence, we also checked the BF score to correctly evaluate the performance. Although there are several references to regulating a good BF score, the correct regulation of a good BF score is still challenging to achieve owing to different key factors according to the study. The BF score is normally classified as reasonable (0.5 ≤ BF < 0.8), very good (0.8 ≤ BF < 0.9), and perfect (0.9 ≤ BF < 1.0)^29–36^. If this regulation is applied to this study, good average contour-matching scores for image segmentation are reported for most of the classes.

As shown in Fig. 1d, afew frequencies were counted from Tibia 14 to Tibia 23 and from Fibula 8 to Fibula 12 because of fewer data than other classes. Such unbalanced data are a common issue in semantic segmentation^11–16^. To resolve this issue, two representative methods were used: partial data augmentation and the addition of weights for classes using the median frequency. In this study, both methods were already applied to resolve the class imbalance, and partial data augmentation, except for the overall data augmentation for all classes, was performed by adding the image and mask data, which involved a selective class from tibia 14 to tibia 23 and from fibula 8 to fibula 12. Although these are general methods for improving the performance of semantic segmentation, several trials were required to optimize the balance in this study. When this balance is maintained under good conditions, such as the amount of data and the number of classes, relatively good performance of the segmentation model can be demonstrated using a small amount of data^11–16^.

However, the deep learning in this study had clear limitations in some cases, as shown in Fig. 4. Under/overestimation by segmentation is the most frequent issue and is caused by the lack of effective data^26–31^. Moreover, the noise issue is related to the overestimation by segmentation. Under/overestimation may not be a major issue for identifying fractured fragments according to the surgeon; however, this issue can inevitably induce low-quality results for reduction simulation using this 3D image or the modeling of a patient-specific device^40,42^. Extreme cases require an additional manual process to edit the results. In this case, it cannot be the realization of the fully automatic process by deep learning.

Another limitation is the weak consistency of the data for the region of spongy bone. The proposed deep learning model in this study predicted a relatively wide area in the region of the spongy bone. The manual masking for the CT image was basically done by using the threshold of the HU. The variation of the threshold for the HU caused the variation of the mask. Especially, although the mask in the region of spongy bone very sensitively changed with the slight difference of the HU, the anatomical attributes for the cortical bone and even spongy bone should be reflected at the mask. In order to overcome these limitations, the best way is to use more high-quality data. Firstly, a lot of data can clearly lead to the high accuracy of deep learning. To achieve this, we need to gather more data from more institutes. Second, the consistency of the data should be maintained at a regular level. The reason for the wide prediction for the region of spongy bone by the deep learning in this study was weak consistency. We need to maintain as much data consistency as possible, including even the region of spongy bone. Lastly, the optimization of the model by adding the data is essential through the adjustment of the hyperparameters. The current hyperparameters for our deep learning model cannot ensure performance when more data is added.

The core point of clinical support ability in the results section is the recognition of fractured fragments within a short time under actual clinical conditions^41–46^. Recognition of fractured fragments is defined as the ability to classify the object class, such as the number of fragments, and it is a lower-level concept than identification, which is the ability to describe the object in detail, such as the size of the fragment and its location and shape. Naturally, deep learning performs the identification step and provides a full 3D reconstructed image for each segmented fragment with classification. The experts performed a recognition step to identify the fractured fragments by scrolling the slice and rotating, expanding, and panning the 3D images. This study required much more observation time than expected. Moreover, when the image quality is low owing to low resolution, artifacts, etc., the recognition time is longer. As a result, deep learning showed significance at almost the same level (from Table 3) with five to eight times faster speeds.

In conclusion, this study demonstrated the good performance of automatic segmentation of inconsistent fractured fragments of the tibia and fibula from CT images using the DeepLab v3+ -based deep learning model. When this model is applied to preoperative surgical planning for trauma surgery such as virtual reduction, it will provide several clinical benefits to the surgeons as well as the patients who suffer from trauma.

## Methods

### Ethics approval and consent to participate

All methods in this study were performed in accordance with relevant guidelines and regulations by the Clinical Trial System of the Catholic Medical Center (CTSC) in the Catholic University of Korea. All experimental protocols were approved by the Institutional Review Board (IRB) at the Seoul St. Mary’s Hospital, the Catholic University of Korea (approval number: KC20RISI1034). Informed consent was obtained from all subjects (and/or their legal guardians) involved in this study.

### Preparation of data

All data for training was collected from one institute; Seoul St. Mary's Hospital in the Catholic University of Korea. The collection source for the data was limited to one device CT scanner (Siemens, SOMATOM Definitions AS+, Munich, Germany) to maintain as much consistency of data as possible. The preparation of data for training has three steps in this study. The first step is the generation of the ground truth as the masking image. For the annotation work for masking image, three experts have conducted it simultaneously using the collected data. Moreover, two orthopaedic specialists have evaluated the results of the annotation. The masking works for the ground truth has essential rules for good performance of the deep learning model. The mask with specific color should cover the only bone regions according to HU value of the CT images, each fractured fragments should be individually separated as the different color masks^37–43^. There are 38 classes and each class has its own mask color with label (Background, Patella, Femur, Tibia from 1 to 23, Fibula from 1 to 12). In the cases of the tibia and fibula, the class was assigned in order of the location of the fragments (from proximal to distal region). Second step is the data augmentation for both CT images and ground truth. Basically, the data augmentation was progressed for all the data to improve the performance of the deep learning^35^. However, in order to maintain data balance for the pixel according to the class, the partial data augmentation is additionally performed by adding the image and mask data which involve selective class from Tibia 14 to Tibia 23, and from Fibula 8 to Fibula 12. The data augmentation basically employed the 2D affine transformation for both the CT images and the labeled mask. Although there are several options for the affine transformation, such as translation, rotation, shear, scale, and reflection, only random translation (X, Y) within 10 pixels and random horizontal reflection were employed for the data augmentation in this study to preserve the original image variable of CT images. Moreover, the data's frequency balance relies on class weighting, determined by the ratio between the median total pixel frequency and the frequency of each pixel for a specific class. Where 'frequency' equals the count of pixels for each class divided by the total pixels in the image, and 'class weight' equals the median total pixel frequency divided by each pixel frequency. Then, the weights according to class were applied to the pixel classification layer in the deep learning model. According to this principle, a few frequencies in the data balance could be compensated to maintain a relatively regular balance. The third step is the composition of the data storage after data normalization. The data storage is database to train the deep learning model. And it contains both the set of CT images and the set of ground truth which is the masked image for the all fragments along the color (Supplementary Fig. 1). In addition, the data was normalized by the range of the RGB to prevent the data bias through the image processing^25–30^. The ratio for training, validation, and test was 60%, 20%, and 20% of the whole data in the data storage. The test data in the data storage was used for evaluating the deep learning models using metrics information after training. And individual 50 CT series as another test group were used for evaluating clinical support ability and showing the intuitive performance of the best deep learning model in this study. The CT series in this group have selected as the case including trauma fracture (with the number of fragments ≤ 13) by the orthpaedic surgeon who did not attend to the evaluation for the clinical support ability of the deep learning.

### Model construction and training

The construction of deep learning models in this study has been progressed by using MATLAB (2022b, Mathworks, USA, MA). And hardware for training models used 2.10 GHz dual Intel(R) Xeon(R) Silver processors 128 GB RAM, and two GPUs as NVIDIA GeForce RTX 3090 with 24 GB GPU memory. The basic encoder-decoder model was selected as DeepLab v3+ due to the performance at the semantic segmentation^18^. Basically, because the DeepLab v3+ shows good performance for the semantic segmentation, the design of the encoder model in the DeepLab v3+ was considered to enhance the strong points of the segmentation for the inconstant fragments from the CT images^18–22^. However, because the position of the optimal depth is hard to be known, we designed the three kinds of the candidate models for the segmentation of the fractured fragments. The Model 1 and Model 2 were based on the ResNet-18 and ResNet-50. And the Model 3 used the structure of Inception-ResNet-v2 as the basic frame^23,24,30,31^. And we have changed the structure of the skip connections and their numbers according to increase of the number of the class. The models have 100 (Model 1), 206 (Model 2), and 853 layers (Model 3) with 113, 227, and 956 connections, respectively. Obviously, the final models are results by a lot of optimization processes through model and hyperparameter tunning. And the best model was the Model 2 when the same data was trained. The final network architecture for Model 2 and its analysis of the network model are shown at the Supplementary Figs. 2, 3, 4, 5. The Model 2 used the solver as the Stochastic Gradient Descent with Momentum with 0.9 momentum. The initial learning rate was 0.0001 with the set of drop period for learning rate, the drop period for learning rate which is epoch period to lower the learning rate was 10 with 0.1 drop factor. Although the initial max epoch was set at 100 with the minibatch size was set at 128, the learning was completed with 63 epochs.

### Evaluation and analysis with statistics

In order to evaluate the performance of the deep learning model for the segmentation of the fractured fragments, we performed the representative two kinds of evaluations for the deep learning models as the metrics model and the clinical support ability. The metrics model has several major indicators. The major indicators are the accuracy for the classified pixel, the intersection over union (IoU), and the boundary F1 (BF) score^25–28^. The accuracy score is basically defined as (True Positive/(True Positive + False Negative)). The accuracy includes the ‘Global Accuracy’ which means the ratio of correctly classified pixels to total pixels, regardless of class, and ‘Mean Accuracy’ for the ratio of correctly classified pixels in each class to total pixels, averaged over all classes. When there is an overwhelming number of classified pixels from a specific class, this major class can lead the an overwhelmingly high Global Accuracy. For this reason, the accuracy of the model for individual classes can be considered with the impact of the major class by comparing it with the Mean Accuracy. In the cases of the ‘IoU’, the score was defined as (True Positive / (True Positive + False Positive + False Negative)). The ‘Mean IoU’ is the IoU of all classes, and we also checked the ‘Weighted IoU’ which is the average IoU of all classes, weighted by the number of pixels in the class^25–28^. Lastly, ‘Mean BF Score’ showed the average contour matching score for image segmentation. The BF Score measures how close the predicted boundary of an object matches the ground truth boundary. Basically, the equation of the BF score is the same as the equation of the Dice Score (2 × precision × recall/(recall + precision)). The precision means the ratio of the number of points on the boundary of the estimated segmentation which are close to the boundary of the ground truth to the length of the estimated boundary^25–28^. The recall is the fraction of True Positives that are detected rather than missed. And the three representative evaluation indicators (‘Accuracy’, ‘IoU’, and ‘Mean BF Score’) according to the class for Model 2 were investigated.

The second evaluation was clinical support ability of the deep learning for the recognition of the fractured fragments comparing with the recognition ability of the specific experts which are highly trained for recognizing the fragments from the CT images^41,42^. For this test, we used the independent 50 CT series which are unrelated to the training. The three experts including data engineer (over 8 years experiences), orthopaedic surgeons (over 10 years experiences) performed the recognition of the fragments using the only 2D/3D CT images and counted the number of the fragments with their approximate shapes by the scrolling the slice of 2D CT images, and the rotating, expanding, panning the 3D CT images. And we recorded the time for recognizing the fractured fragments except the loading time for 2D or 3D image view. Whereas the deep learning performed the automatic segmentation along each fragment from the CT series and reconstructed 3D images showing the segmented fragments. And we calculated the statistics as the paired t-test between two groups including the number of counted fragments by the experts and deep learning to check the significance.

### Supplementary Information

Supplementary Figures.

## Data Availability

The authors declare that partial data (68 CT images with ground truth from hospital) was already uploaded in the Github account of D.Y. who is a corresponding author (link: https://github.com/Louis-Youn/Code_Storage). The data has been anonymized and the personal information has been removed. Because the full datasets are still protected by the privacy issues and regulation policies, additional data to train or test model can be acquired by contacting to corresponding author (D.Y., E-mail: louis_youn@kavilab.ai).

## Code availability

The source code which was employed for this study is available by downloading from Github account of D.Y. who is a corresponding author (link: https://github.com/Louis-Youn/Code_Storage). The source code should be used for academic research only.
