# Supplementary material for: Automatic segmentation of inconstant fractured fragments for tibia/fibula from CT images using deep learning
Source: Sci Rep. 2023 Nov 22;13:20431. doi: 10.1038/s41598-023-47706-4 (PMC10665312; doi:10.1038/s41598-023-47706-4)
Supplement: Supplementary file 1 — Supplementary Figures. [file 41598_2023_47706_MOESM1_ESM.docx]

**Supplementary Information**

**Automatic Segmentation of Inconstant Fractured Fragments for Tibia/Fibula from CT images using Deep Learning**

**Hyeonjoo Kim^1, 3),^ Young Dae Jeon^2)^, Ki Bong Park^2)^, Hayeong Cha^3)^,**

**Moo-Sub Kim^3)^, Juyeon You^3)^, Se-Won Lee^4)^, Se-Won Lee^4)^, Seung-Han Shin^5)^,**

**Yang-Guk Chung^5)^, Sung Bin Kang^3)^, Won Seuk Jang^1, *^, Do-Kun Yoon^3, *^**

*^1^Department of Medical Device Engineering and Management, College of*

*Medicine, Yonsei University, Seoul, Republic of Korea*

*^2^Department of Orthopedic Surgery, University of Ulsan, College of*

*Medicine, Ulsan University Hospital, Ulsan, Republic of Korea*

*^3^Industrial R&D Center, KAVILAB Co. Ltd., Seoul, Republic of Korea*

*^4^Department of Orthopedic Surgery, Yeouido St. Mary’s Hospital,*

*College of Medicine, The Catholic University of Korea, Seoul, Republic of Korea.*

*^5^Department of Orthopedic Surgery, Seoul St. Mary’s Hospital,*

*College of Medicine, The Catholic University of Korea, Seoul, Republic of Korea.*

Conflicts of Interest: None

^*^Author to whom correspondence should be addressed.

Do-Kun Yoon, Ph.D. and Won Seuk Jang, Ph.D.

E**−**mail: louis_youn@kavilab.ai

Phone: +82-2-525-1109


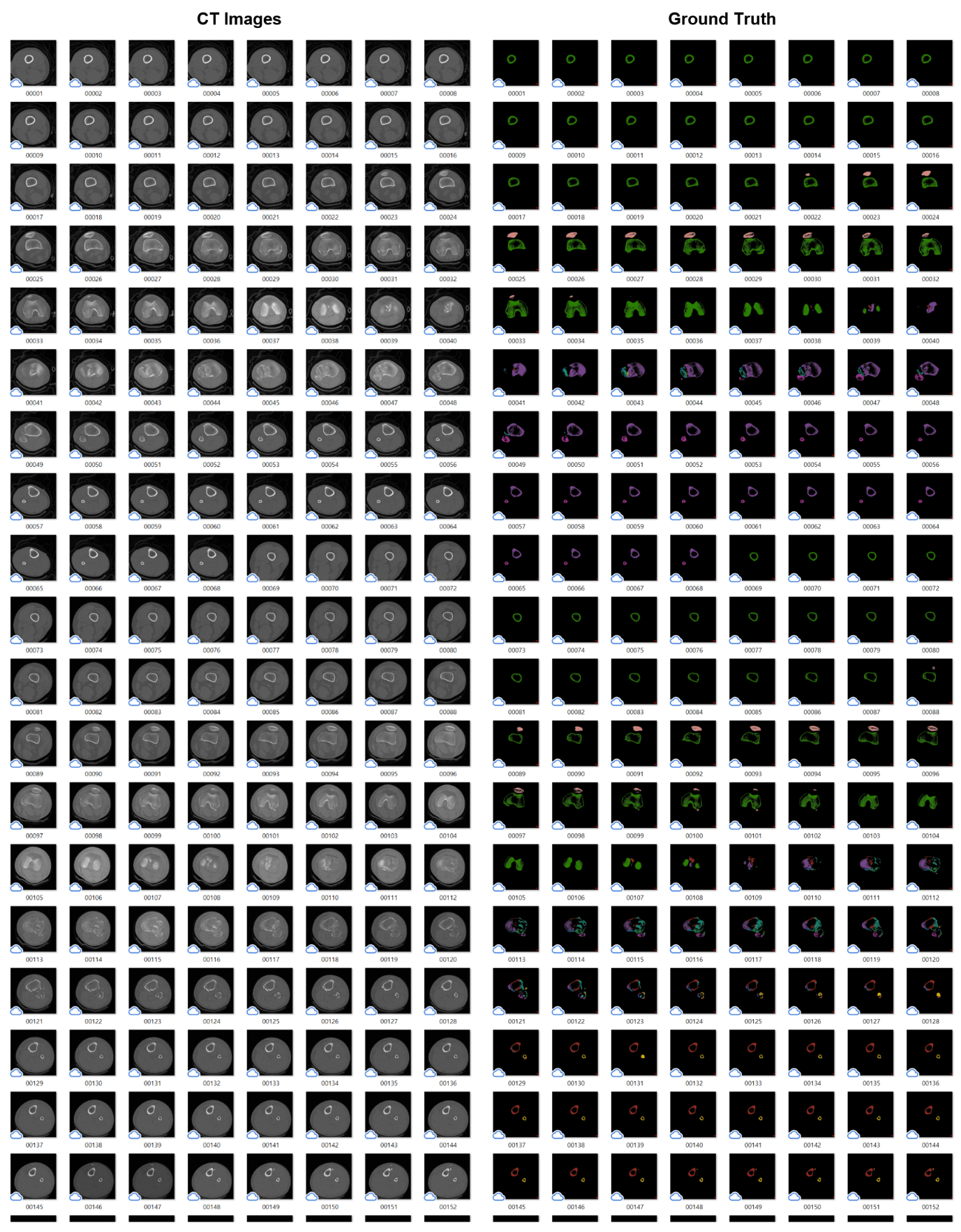


**Supplementary Figure 1 Example of training data for network model in this study.** The left side shows the original CT images which were converted to the PNG format. The right side shows the mask images that mean the ground truth in this study.


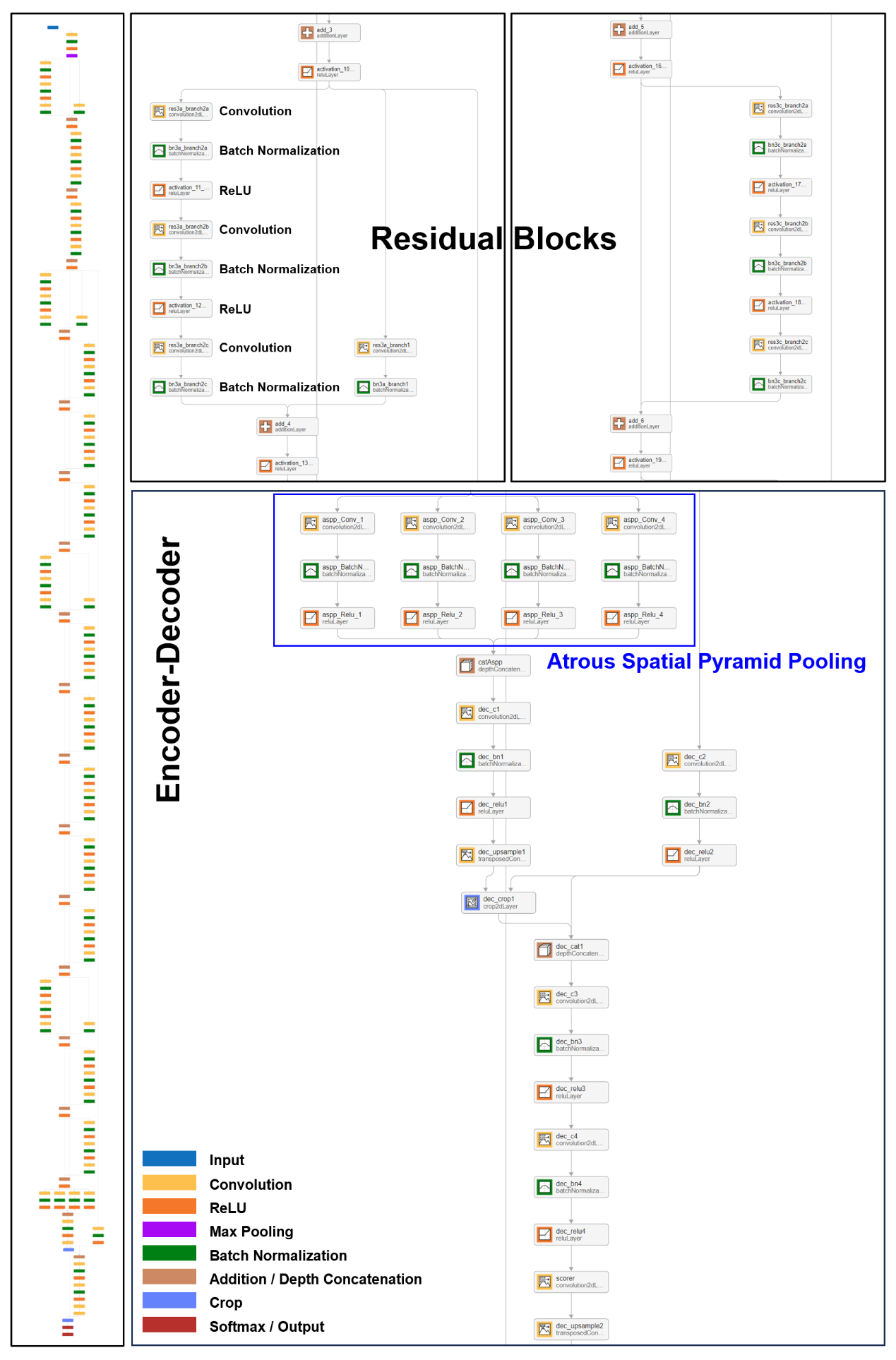


**Supplementary Figure 2 Detail structure of segmentation network model employing the DeepLab v3+ model.** The encoder was composed of the repetition of residual blocks, the decoder involved the Atrous spatial pyramid pooling.


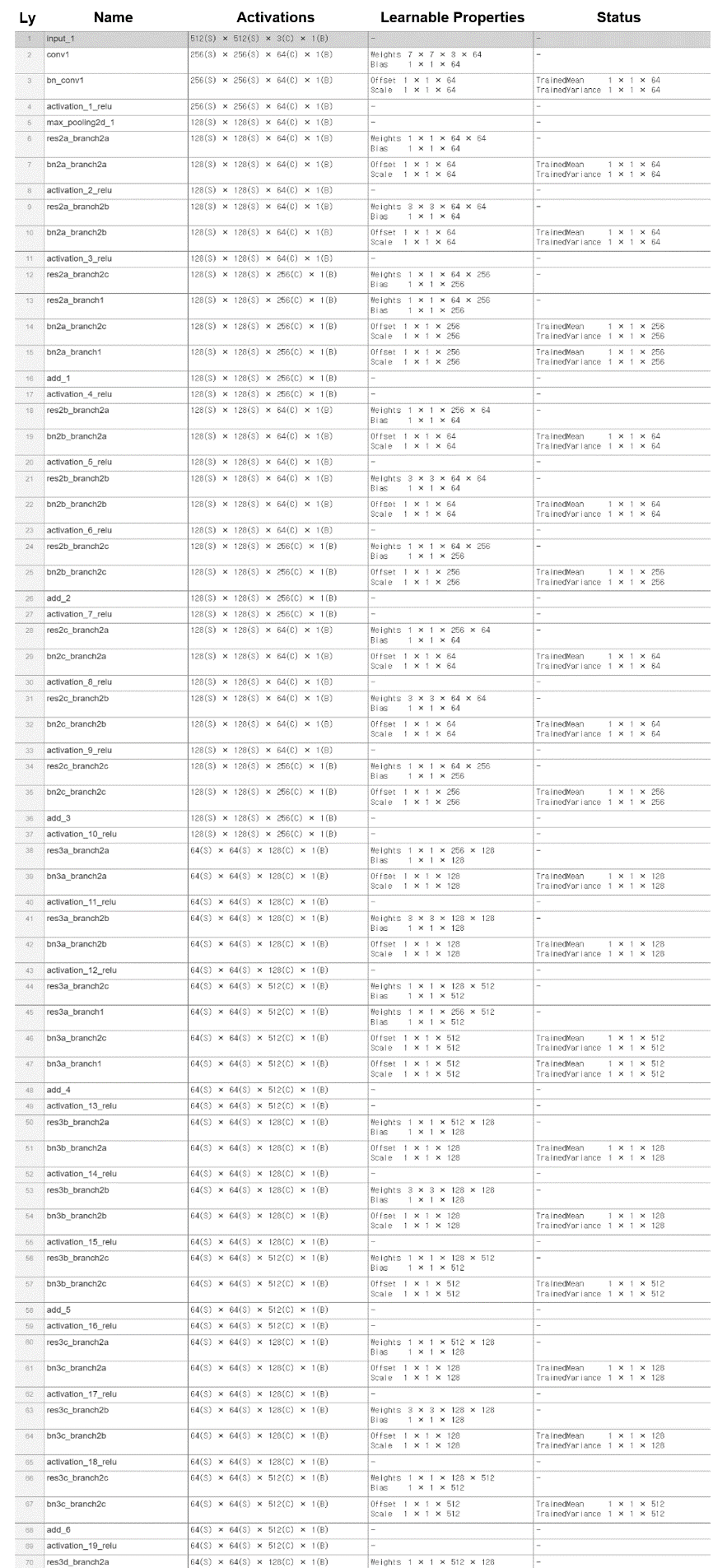


**Supplementary Figure 3 Statement and analysis as layer by layer (1-70th) for deep learning model for this study.**


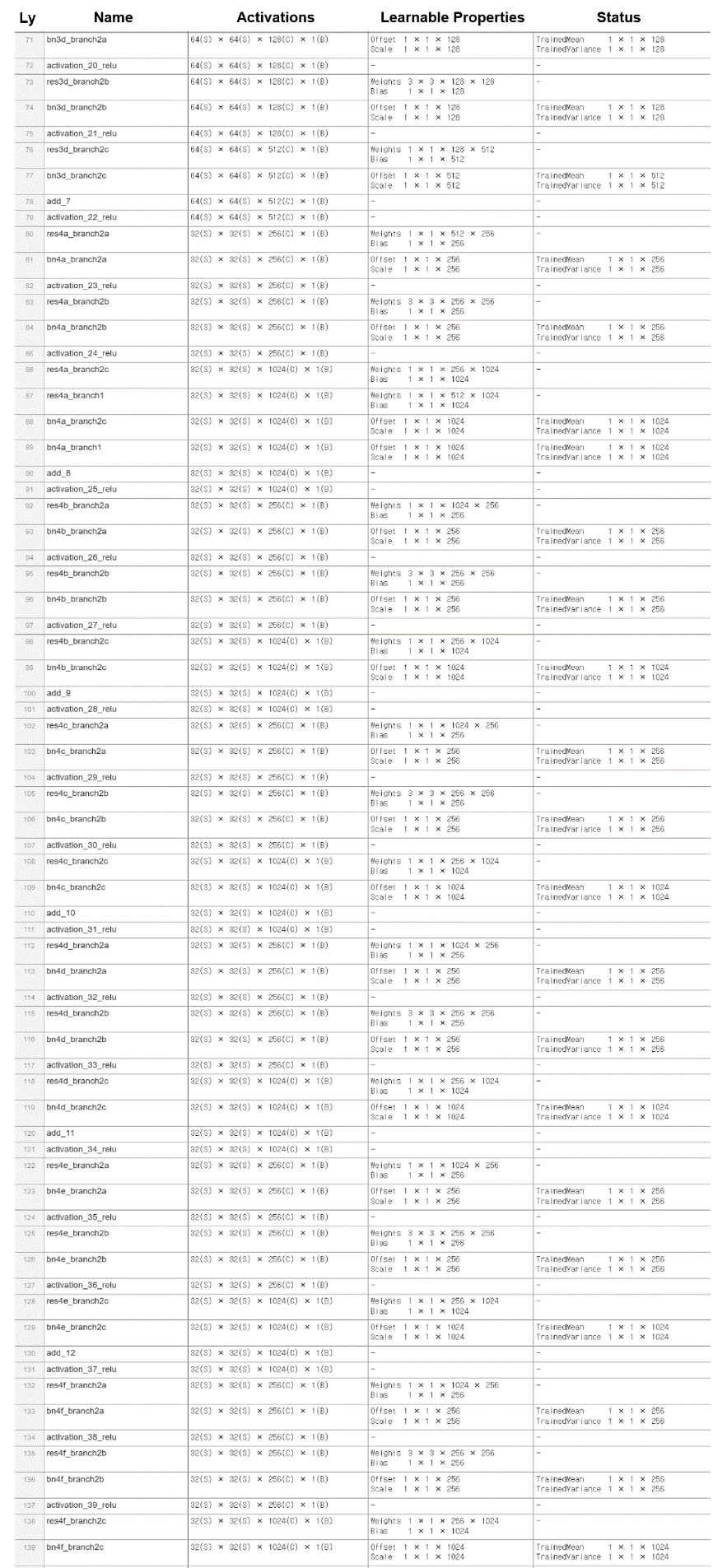


**Supplementary Figure 4 Statement and analysis as layer by layer (71-139th) for deep learning model for this study.**


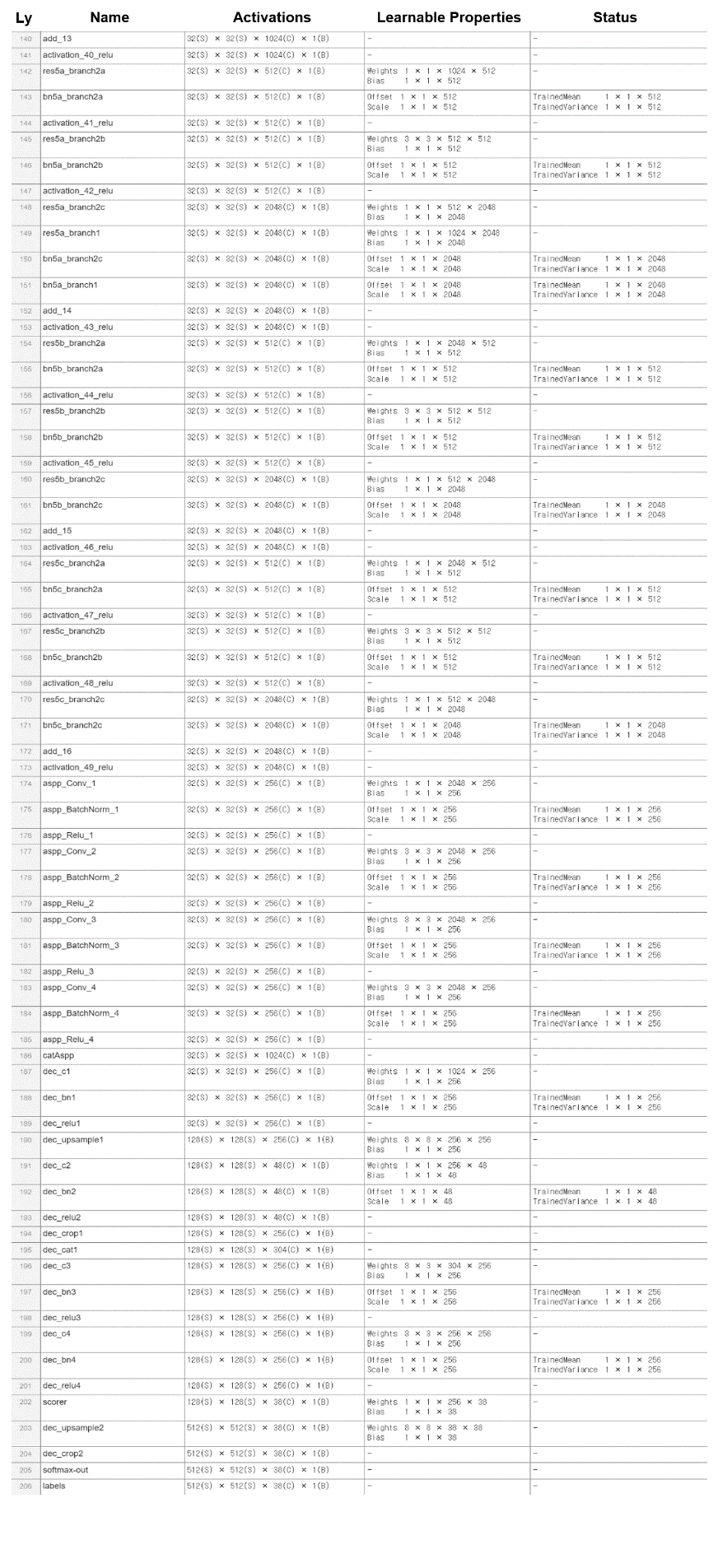


**Supplementary Figure 5 Statement and analysis as layer by layer (140-206th) for deep learning model for this study.**
